# Fistula incidence after primary repair and correlation with cleft width-to-palatum width ratio: A prospective cohort study

**DOI:** 10.1016/j.amsu.2021.102183

**Published:** 2021-02-23

**Authors:** Rosadi Seswandhana, Firdian Makrufardi, Gentur Sudjatmiko

**Affiliations:** a Plastic, Reconstructive, and Aesthetic Surgery Division, Department of Surgery, Faculty of Medicine, Public Health and Nursing, Universitas Gadjah Mada/Dr. Sardjito Hospital, Yogyakarta, 55281, Indonesia; b Department of Plastic Reconstructive, and Aesthetic Surgery, Faculty of Medicine, Universitas Indonesia/Dr. Cipto Mangunkusumo Hospital, Jakarta, Indonesia

**Keywords:** Primary palatoplasty, Two-flap three layers suturing technique, Cleft width and palate width ratio, Fistula occurrence

## Abstract

**Background:**

Cleft lip with or without cleft palate is one of the most common birth defects and is certainly the most visible. Fistula rate after primary palatoplasty was ranging between 10 and 23% and could be detected in the first three weeks after surgery. The cleft width is the frequent factor which was assumed to correspond to fistula occurrence. This study aimed to find correlation between fistula occurrence with cleft width and palatum width ratio after primary palate repair.

**Methods:**

A prospective cohort study was conducted on 16 subjects, which consisted of 10 males and 6 females. We measured width of cleft palate, width of rest palate and width of palate arch on three level measurements (posterior, junction and anterior). The surgery was performed using the two-flap and three layers suturing technique.

**Results:**

Sixteen patients were enrolled in this study during January and February 2008 . Ten patients were diagnosed with unilateral cleft palate while six patients had bilateral cleft palate. Mean of age was 22.31 ± 5.86 month. Correlation analysis between fistula occurrence and cleft width, cleft width-remnant palate width ratio and cleft width-palate arch width ratio using logistic regression did not show statistical correlation, and the same result was found between fistula occurrence and hemoglobin level, white blood count, nutritional status, cleft type and caries dentis factors (*p* > 0.05).

**Conclusion:**

Width of the cleft is not a factor associated with fistula occurrence. Two-flap three layers technique could be considered as a simple technique and gives a low rate of fistula occurrence.

## Highlights

• Cleft lip is one of the most common birth defects and is certainly the most visible.
• Two-flap three layers technique gives a low rate of fistula occurrence.
• Two-flap three layers technique could be considered as a simple technique.

## 1 Introduction

Cleft lip with or without cleft palate is one of the most common birth defects. Incidence varies among ethnic groups, ranging from 3.6 per 1000 live births among Native Americans to 2.0 per 1000 among Asians [1]. Results from a large study in Iran that supports global statistics showed that cleft lip is twice as common among boys, and cleft palate more frequent among girls [2].

In Indonesia, primary palatoplasty is performed as a second stage reconstruction of a cleft lip and palate. In general, this procedure is conducted during the second year of life, with the average of 1.5 year of age. Timing is based on when the child starts to speak actively and to avoid growth disturbance of the maxilla [3].

The broad goal of cleft palate treatment is to separate the oral and nasal cavities. Although this is not absolutely necessary for feeding, it is advantageous to normalize feeding and decrease regurgitation and nasal irritation. Repositioning of the soft palate musculature to anatomically recreate the palate is essential and necessary to establish normal speech. Another goal of palate repair is to minimize restriction of growth of the maxilla in both sagittal and transverse dimensions [4].

The best time to surgically close the cleft palate is when the ratio of the posterior cleft to the complete palatal surface medial to the alveolar ridges is not more than 10% [5]. Pre-surgical orthopedics should not promote palatal development above its natural growth capacity. Extensive watch flaps with or without palatal push-back surgery are available [23].

Palatal fistulae are an early adverse reaction to primary palate repair. Before the 21st century, the frequency of fistulae after primary palatoplasty was reported to be about 12–45% [6]. Fistulas are more common in greater clefts [7]. This study aimed to identify any correlation between fistula occurrence with cleft width and palatum width ratio after primary palatoplasty.

## 2 Material and methods

### 2.1 Patients

**Fig. 1:**
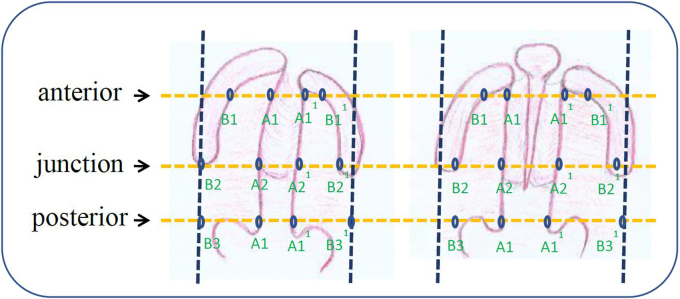
Cleft width: A-A^1^; Remnant plate width: A-B + A^1^-B^1^; Palatal arch width: B-B^1^. Cleft width-to-Remnant palate ratio = (A-A^1^)/(A-B + A^1^-B^1^). Cleft width-to-Palatal arch width = (A-A^1^)/(B-B^1^).

The prospective cohort study included 16 participants, consisting of 10 males and 6 females. We included patients who admitted in our tertiary hospital from January to February 2008. Under anesthesia prior to surgery, we weighed and measured the large cleft palate, large rest palate and wide palate arch on three level scales (posterior, junction and anterior) using a metal caliper and ruler (see Fig. 1).

The diagnosis of cleft lip with or without cleft palate in our institution was established using clinical manifestations and imaging [20]. The body weight by age z (WAZ) anthropometry was classified into four categories as follows: a) severely underweight, b) underweight, c) normal, and d) overweight [21].

Written informed consent was obtained from all parents for joining this study. This study has been reported in line with the Strengthening the Reporting of Cohort Studies in Surgery (STROCSS) criteria [19].

### 2.2 Surgical technique and outcomes

The surgery was performed using a two-flap design, and for anesthesia, 0.5% lidocaine with epinephrine 1:200,000 were injected in and around the palatal defect and the lateral shelves of the palate.

After surgery, all of the patients were hospitalized for 1 up to 2 days. Amoxicillin 10 mg/kg body weight and paracetamol 10 mg/kg body weight were given 3 times a day for 7 days. Follow-up was performed every week postoperatively in the outpatient clinic for the first three weeks, and then every month (February 2018–December 2018). At each visit, clinical examination was performed, and every fistula occurrence was noted. All of the following data were collected: (1) Demographic characteristics, (2) results of preoperative findings, and (3) postoperative fistula occurrences.

### 2.3 Statistical analysis

Categorical variables are presented as counts and percentages. Continuous data are presented as mean and standard deviation (SD) for normally distributed data or median for skewed data. Logistic regression was performed on patients undergoing surgery, with observation time starting at the time after surgery. We correlated our patients’ data using spearmen correlation test with p < 0.05 considered as significant. Data were analyzed using IBM SPSS Statistics 23rd version (IBM Corp., Chicago).

## 3 Results

Sixteen patients were enrolled in this study during January and February 2018. The male-to-female ratio was 1.6:1, and the age of the patient ranged from 14 to 39 months (22.31 ± 5.86 months). Ten patients were diagnosed as unilateral cleft palate while 6 patients were bilateral cleft palate.

Characteristics of patients are shown in Table 1. Four patients suffered moderate and mild anemia (hemoglobin level between 8 and 10.9 g/dL), and mean of hemoglobin level was 11.46 g/dL (±1.20). Mean of white blood count was 9500 (±2515.55)/mm^3^. Mean of the body weight was 10.18 ± 0.32 kg, with mean of WAZ anthropometry value of 1.66 ± 1.22. Two patients were indicated as severe underweight (WAZ anthropometry value ≤ −3.00), 5 patients were underweight (z anthropometry value −2.99 to −2.00), and 9 patients were normoweight (WAZ anthropometry value −1.99–1.99). Three patients (18.75%) had caries on their teeth.

**Table 1:** Demographic characteristics of patients.

Variables	Results
Patient (N)	16
Age Range Mean (SD)	14–39 months 22.31 (±5.85) months
Sex Male Female	10 6
Type of the cleft Unilateral Bilateral	10 patients (62.50%) 6 patients (37.50%)
Hemoglobin level, mean (SD)	11.46 (±1.20) g%
White blood count, mean (SD)	9500 (2515.55)/mm^3^
Body weight, mean (SD)	10.18 (±1.32) kg
Body weight by age z anthropometry, mean (SD) Normoweight (−1.99 – 1.99) Underweight (−2.99 to −2.00) Severe underweight (≤−3.00)	−1.66 (±1,22) 9 patients (56.25%) 5 patients (31.25%) 2 patients (12.50%)
Caries dentis	3 patients (18.75%)

SD: Standard deviation.

In the anterior area, width of cleft palate ranged from 6 to 14 mm (9.69 ± 2.35). Cleft palate width ranged from 8.5 to 20 mm (13.50 ± 2.94) in the junction area, while in posterior area ranged from 10 to 21 mm (13.38 ± 3.11).

Width of both remnant palate in the anterior area ranged from 15 to 29 mm (21.53 + 3.96). In the junction area, minimal remnant palate width was 20 mm and maximal was 29.5 mm (25.25 ± 3.45). Meanwhile, in the posterior area, remnant palate width ranged from 20 to 31 mm (26.65 ± 3.18).

Width of palate arch in the anterior area ranged 24–37 mm (31.22 ± 4.04). In the junction area, minimal palate arch width was 32 mm and maximal was 44 mm (38.75 ± 4.04). In the posterior area, palate arch width ranged from 31 to 47 mm (39.94 ± 4.40) (Table 2).

**Table 2:** Width of cleft, palate and palate arch measurement results.

Measurements (in mm)	Minimum	Maximum	Mean	SD
Anterior cleft width	6.0	14.0	9.687	2.3514
Junction cleft width	8.5	20.0	13.500	2.9439
Posterior cleft width	10.0	21.0	13.375	3.1118
Anterior remnant palate width	15.0	29.0	21.531	3.9643
Junction remnant palate width	20.0	29.5	25.250	3.4496
Posterior remnant palate width	20.0	31.0	26.562	3.1774
Anterior palate arch	24.0	37.0	31.219	4.0372
Junction palate arch	32.0	44.0	38.750	4.0373
Posterior palate arch	31.0	47.0	39.937	4.4041

mm, millimeters; SD, standard deviation.

Minimal ratio cleft width-to-remnant palate width was 20.69% in the anterior area and maximal ratio was 90,91% in the junction area, likewise minimal ratio cleft width-to-palate arch was 17.14% and maximal was 47.62% in the junction area (Table 3).

**Table 3:** Cleft width-to-remnant palate width ratio and cleft width-to-palate arch width ratio.

Ratio (%) in millimeters	Minimum	Maximum	Mean	SD
Anterior cleft width-to-remnant palate width	20.69	77.78	46.7865	14.89150
Junction cleft width-to-remnant palate width	29.31	90.91	54.7579	15.37502
Posterior cleft width-to-remnant palate width	33.33	80.77	51.0406	12.93127
Anterior cleft width-to-palate arch width	17.14	43.75	31.2125	6.99950
Junction cleft width-to-palate arch width	22.67	47.62	34.7940	6.38020
Posterior cleft width-to-palate arch width	25.00	44.68	33.3497	5.54421

SD, standard deviation.

In the first three weeks observation after primary palatoplasty, we found only 1 fistula occurrence among 16 patients (6.25%) in the junction area. The size of fistula was about 2 mm, which was observed in day 18th after surgery.

Correlation analysis between fistula occurrence and cleft width, cleft width-remnant palate width ratio and cleft width-palate arch width ratio using logistic regression did not show statistical correlation, and the same result was found between fistula occurrence and hemoglobin level, white blood count, nutritional status, cleft type and caries dentis factors (Table 4).

**Table 4:** Correlation analysis between fistula occurrence and cleft width, cleft width-to remnant palate width ratio and cleft width-to-palate arch width ratio in millimeters.

Logistic Regression	N	Fistula Occurrence	*Odds Ratio*	95% *Confidence Interval*	
Cleft width	16	1	0.93701	0.46312	1.89581
Cleft width-to-remnant palate width ratio	16	1	1.00446	0.87516	1.15286
Cleft width-to-palate arch width ratio	16	1	0.99425	0.71599	1.38064
Haemoglobin level	16	1	0.82052	0.12976	5.18844
White blood count	16	1	1.00034	0.99937	1.00130
Nutritional status	16	1	1.17662	0.20171	6.86339

Spearman correlation between cleft type and fistula occurrence: 0.488 (*p*-value: 0.055).

Spearman correlation between cleft type and fistula occurrence: 0.220 (*p*-value:0.410).

## 4 Discussion

In this study, male-to-female ratio (1.6:1) was appropriate with cleft lip and palate demographic data in general [8]. Average of patients age was about 22.31 ± 5.86 month which was when they start to actively verbalize. In cleft lip and palate, the timing for closing a cleft palate has traditionally been based on the age of the patient and the onset of speech (usually between 6 and 8 months), irrespective of the physical assets and defects of the affected tissue [5]. On the other hand, many surgeons are apprehensive about maxilla growth disturbance if the surgery is done in the early period of life. Surgeons have decided that timing of closing hard palate was more conservative lately after primary soft palate reconstruction [4]. A report found maxilla growth disturbance in early palatoplasty and suggested timing of palatoplasty ranged from 4 to 6 years [1]. Another study reported there is an intrinsic tissue deficiency in all groups of patients with cleft. However, the sagittal development is still comparable to that of a normal population [9].

According to postoperative analysis, there were no correlations between age, sex, hemoglobin level, white blood count, nutritional status, cleft type and caries dentis status with fistula occurrence. A study found a similar result that age, sex, body weight, and hemoglobin level were not correlated to fistula occurrence [10]. In this study, the nutritional status was not only based on body weight but also on WAZ.

The widest cleft was found on the junction area between hard and soft palate (13.50 ± 2.94 mm) while the narrowest was on the anterior area (9.68 ± 2.35 mm). The widest remnant palate was on the posterior area (26.56 ± 3.17 mm), while the narrowest was on the anterior area (21.53 ± 3.96 mm). The widest arch of the palate was on the posterior area (39.93 ± 4.40 mm) while the narrowest was on the anterior area (31.22 ± 3.17 mm). A report showed average cleft width was 12.22 mm (range from 8 to 15 mm). The average age at the time of closure was 11.75 months (range from 8 to 28 months) [7]. Anterior palatal volume-to-total palatal volume ratio was lesser in the cleft palate group compared to the non-cleft group, but there was significant difference in width-to-length palatal ratio between cleft and non-cleft groups [11].

In this study, we found that fistula occurred only in 1 among 16 patients (6.25%). These results show a low fistula rate compared to many studies (before year 2000) that ranged from 12% to 45% [[12], [13], [14]]. Whereas fistula rate ranged from 5 to 33% as reported in studies after the 20th century [6,14,15]. In general, fistula post-palatoplasty using all types of techniques ranged from 0 to 50%; but most studies reported the incidence of fistula ranged from 11 to 25%. In recent times, fistulae are likely to be more prevalent in wider clefts and dependent on type of repair. The site most likely to fistulize is the closure site, where the soft-palate and hard-palate junction occurs [7]. A study using the two-flap technique had fistula rate of about 8.6% [6].

Correlation analysis between fistula occurrence and cleft width, cleft width-remnant palate width ratio and cleft width-palate arch width ratio using logistic regression did not show statistical correlation. A study in 1997 showed there was no correlation between cleft width and fistula occurrence [12], while another study showed that fistula rate increased when the palate cleft was more severe according to the Veau classification [6].

In our study, palatoplasty was conducted using two-flap three layers suturing. Another study using the two-flap technique reported 3.4% of fistula rate [14]. A study reported the Wardill Kilner technique had fistula rate that was higher than Von Langenback or Dorance technique [16], while one study reported the two-flap technique had fistula rate lower than the Von Langenback technique [12]. The Furlow technique using decellularized dermis had a fistula rate of about 3.2% [7].

Three layer suturing technique was more intended to reach satisfying long term velopharyngeal function result. A study reported satisfying long term velopharyngeal competence result using three layers suturing, but this technique had fistula rate higher than the two layers suturing [22].

A study reported that palatoplasty technique has evolved over a 24-year period and appeared to be a significant reduction in velopharyngeal incompetence associated with increased radical surgery and experience of the operator. More radical muscle dissection and retropositioning have generally improved palatal function, but the search continues for more functional palate repair [17].

Prophylactic Gentamycin® injection that was used in this study was based on the last protocol of our operating room. A study reported a lack of consensus and wide disparity among centers. The most popular antibiotics were co-amoxiclav, phenoxymethylpenicillin, or flucloxacillin and ampicillin combined. A study showed that some random control clinical trials are needed to establish national recommendations for the rational use of prophylactic antibiotics in primary cleft surgery [18].

Our study has limitation related to time of the study and further larger study is needed to confirm our findings. Moreover, there might be a bias in measurement, but we tried to minimize that by adding a second data collector who validated measurement process.

Patients with cleft lip with or without cleft palate in tertiary research hospital are susceptible to have a fistula after primary repair. That might be caused by several factors, one of those factors is the operation technique [14]. Therefore, the choice of surgical technique and follow-up requires careful attention by the physician.

## 5 Conclusions

Width of the cleft is not an associated factor to fistula occurrence. Two-flap three layers technique could be considered as a simple technique and give a low rate of fistula occurrence.

## Consent

Written informed consent was obtained from the parents before joining the study. A copy of the written consent is available for review by the Editor-in-Chief of this journal on request.

## Provenance and peer review

Not commissioned, externally peer reviewed.

## Declaration of competing interest

No potential conflict of interest relevant to this article was reported.

## Registration of research studies

Name of the registry: Research Registry

Unique Identifying number or registration ID: researchregistry3187

Hyperlink to your specific registration (must be publicly accessible and will be checked): https://www.researchregistry.com/browse-the-registry#home/registrationdetails/59f493fec166b04cad144276/

## References

[1] Mulliken J.B. (2004). The changing faces of children with cleft lip and palate. N. Engl. J. Med..

[2] Noorollahian M., Nematy M., Dolatian A., Ghesmati H., Akhlaghi S., Khademi G.R. (2015). Cleft lip and palate and related factors: a 10 years study in university hospitalised patients at Mashhad--Iran. Afr. J. Paediatr. Surg..

[3] Margulis A., Kryger Z.B., Sisco M. (2007). Cleft lip. Practical Plastic Surgery.

[4] Margulis A., Kryger Z.B., Sisco M. (2007). Cleft palate. Practical Plastic Surgery.

[5] Berkowitz S., Duncan R., Evans C., Friede H., Kuijpers-Jagtman A.M., Prahl-Anderson B., Rosenstein S. (2005). Timing of cleft palate closure should be based on the ratio of the area of the cleft to that of the palatal segments and not on age alone. Plast. Reconstr. Surg..

[6] Muzaffar A.R., Byrd H.S., Rohrich R.J., Johns D.F., LeBlanc D., Beran S.J., Anderson C., Papaioannou aA. A. (2001). Incidence of cleft palate fistula: an institutional experience with two-stage palatal repair. Plast. Reconstr. Surg..

[7] Helling E.R., Dev V.R., Garza J., Barone C., Nelluri P., Wang P.T. (2006). Low fistula rate in palatal clefts closed with the Furlow technique using decellularized dermis. Plast. Reconstr. Surg..

[8] Hopper R.A., Cutting C., Grayson B., Thorne C.H., Beasley R.W., Aston S.J. (2007). Cleft lip and palate. Grabb and Smith's Plastic Surgery.

[9] Diah E., Lo L.J., Huang C.S., Sudjatmiko G., Susanto I., Chen Y.R. (2007). Maxillary growth of adult patients with unoperated cleft: answers to the debates. J. Plast. Reconstr. Aesthetic Surg..

[10] Mak S.Y., Wong W.H., Or C.K., Poon A.M. (2006). Incidence and cluster occurrence of palatal fistula after furlow palatoplasty by a single surgeon. Ann. Plast. Surg..

[11] Oyama T., Sunakawa H., Arakaki K., Shinya T., Tengan T., Hiratsuka H., Maekawa T. (2002). Articulation disorders associated with maxillary growth after attainment of normal articulation after primary palatoplasty for cleft palate. Ann. Plast. Surg..

[12] Emory R.E., Clay R.P., Bite U., Jackson I.T. (1997 May). Fistula formation and repair after palatal closure: an institutional perspective. Plast. Reconstr. Surg..

[13] Rohrich R.J., Rowsell A.R., Johns D.F., Drury M.A., Grieg G., Watson D.J., Godfrey A.M., Poole M.D. (1996). Timing of hard palatal closure: a critical long-term analysis. Plast. Reconstr. Surg..

[14] Wilhelmi B.J., Appelt E.A., Hill L., Blackwell S.J. (2001). Palatal fistulas: rare with the two-flap palatoplasty repair. Plast. Reconstr. Surg..

[15] Diah E., Lo L.J., Yun C., Wang R., Wahyuni L.K., Chen Y.R. (2007). Cleft oronasal fistula: a review of treatment results and a surgical management algorithm proposal. Chang Gung Med. J..

[16] Moore M.D., Lawrence W.T., Ptak J.J., Trier W.C. (1988). Complications of primary palatoplasty: a twenty-one-year review. Cleft Palate J..

[17] Sommerlad B.C. (2003). A technique for cleft palate repair. Plast. Reconstr. Surg..

[18] Smyth A.G., Knepil G.J. (2008). Prophylactic antibiotics and surgery for primary clefts. Br. J. Oral Maxillofac. Surg..

[19] Agha R., Abdall-Razak A., Crossley E., Dowlut N., Iosifidis C., Mathew G., STROCSS Group (2019 Dec). STROCSS 2019 guideline: strengthening the reporting of cohort studies in surgery. Int. J. Surg..

[20] Kim D.W., Chung S.W., Jung H.D., Jung Y.S. (2015). Prenatal ultrasonographic diagnosis of cleft lip with or without cleft palate; pitfalls and considerations. Maxillofac. Plast. Reconstr. Surg..

[21] WHO. Training (2008). Course on Child Growth Assessment.

[22] Hassan M.E., Askar S. (2007). Does palatal muscle reconstruction affect the functional outcome of cleft palate surgery?. Plast. Reconstr. Surg..

[23] Alzain I., Batwa W., Cash A., Murshid Z.A. (2017). Presurgical cleft lip and palate orthopedics: an overview. Clin. Cosmet. Invest. Dent..

